# Behavioral Inefficiency on a Risky Decision-Making Task in Adulthood after Adolescent Intermittent Ethanol Exposure in Rats

**DOI:** 10.1038/s41598-017-04704-7

**Published:** 2017-07-05

**Authors:** Kelsey M. Miller, Mary-Louise Risher, Shawn K. Acheson, Matthew Darlow, Hannah G. Sexton, Nicole Schramm-Sapyta, H. S. Swartzwelder

**Affiliations:** 1 Neurobiology Research Laboratory, Durham VA Medical Center, Durham, NC 27705 USA; 2 Department of Psychiatry and Behavioral Sciences, Duke University Medical Center, Durham, NC 27710 USA; 3 Duke Institute for Brain Sciences, Durham, NC 27710 USA; 4 Department of Psychology and Neuroscience, Duke University, Durham, NC 27708 USA; 5 McGovern Medical School, Houston, TX 77030 USA

## Abstract

Adolescence is a period of development in neural circuits that are critical for adult functioning. There is a relationship between alcohol exposure and risky decision-making, though the enduring effects of adolescent ethanol exposure on risky decision-making in adulthood have not been fully explored. Studies using positive reinforcement have shown that adolescent intermittent ethanol (AIE) exposure results in higher levels of risky decision-making in adulthood, but the effects of AIE on punishment-mediated decision-making have not been explored. Adolescent rats were exposed to AIE or saline vehicle across a 16-day period, and then allowed to mature into adulthood. They were then trained to lever press for food reward and were assessed for risky decision-making by pairing increased levels of food reward with the probability of footshock punishment. AIE did not alter punishment-mediated risky decision-making. However, it did result in a significant increase in the delay to lever pressing. This finding is consistent with previous reports, using other behavioral tasks, which show decreased behavioral efficiency in adulthood after AIE. These findings indicate that AIE increases behavioral inefficiency, but not punishment-mediated risk-taking, in adulthood. Thus they contribute to a more nuanced understanding of the long-term effects of AIE on adult behavior.

## Introduction

Adolescence is a time of substantial change in structure and function of the brain. This is especially true for the pre-frontal cortex, limbic system, and hippocampus: brain regions widely considered to be responsible for planning, impulse control, reward, and general decision-making^1, 2^. Ongoing brain maturation occurring within the pre-frontal cortex, throughout adolescence and into the early 20’s in humans, contributes to the increased risk-taking and decreased behavioral inhibition that is common within this age group^3^. It is often hypothesized that adolescent risk-taking, combined with the novelty of drinking, may contribute to frequent or excessive alcohol consumption by adolescents. Nationwide surveys conducted in 2014 reported that 20.8% of U.S. high school students and 39.0% of college students consumed ethanol in a ‘binge drinking’ manner in the past month, where such drinking is defined as the consumption of five or more alcoholic drinks within a couple of hours^4, 5^.

Studies in animal models utilize adolescent intermittent ethanol (AIE) exposure to model the pattern and doses associated with binge drinking in human adolescents. With respect to decision-making specifically, some rodent studies have used the probability-discounting paradigm of risk, which allows animals to choose between a “safe” lever that delivers a small reward 100% of the time and a “risky” lever that presents a larger reward probabilistically. Such studies have shown that risky decision-making in adulthood is higher among animals exposed to AIE than in controls^6–8^. Animals repeatedly exposed to alcohol during adolescence are more likely than controls to choose the “risky” lever in adulthood, even when the larger reward is unlikely^6–9^. Importantly, the increased risky decision-making associated with AIE has not been shown to occur after comparable intermittent ethanol exposure during adulthood^9^. However, probability-discounting tasks assess risk with respect to the potential loss of reward and not with respect to the possibility of punishment, which is a salient motivator for risk avoidance in rodents and humans alike.

Few studies have utilized risky decision tasks in which there is risk of explicit punishment^10, 11^. One such model is Simon’s risky decision-making task (RDT)^12^, in which, like the probability-discounting task, animals are provided with a choice of two levers: the “safe” lever provides a small food reward and the “risky” lever provides a large food reward. However, instead of the large reward being presented probabilistically, the large reward is always provided when the “risky” lever is pressed but is associated probabilistically with footshock. This better simulates the complexity of natural decision-making, because it requires decisions to be made by integrating the impact of rewards and punishments across different modalities rather than with respect to the probability of food delivery alone^12^. Importantly, although the results of Simon’s RDT model significantly correlate with the probability-discounting task, the correlation is relatively weak (r^2^ = 0.35, p < 0.05)^12^. Thus, it is likely that the RDT assesses a combination of risk and reward features that are distinct from those assessed by the probability-discounting task. No studies have assessed the effects of AIE on animals’ response to this particular kind of risk (i.e. one that juxtaposes the risk of explicit punishment with a larger reward). Therefore, we designed the present experiments to assess the effects of AIE on risky decision-making in adulthood, using the punishment-mediated RDT model. Based on previous AIE studies using appetitive risk alone, we would hypothesize that adult animals previously exposed to AIE would manifest a greater propensity toward risky decision making^6–9^.

## Methods

### Apparatus

Behavioral testing took place in Med Associates rat operant chambers (30.5 cm × 24.1 cm × 21.0 cm; St. Albans, VT). Two levers (3 cm × 1.8 cm each, 3 cm above the floor) were mounted on one wall of the chamber. A food trough, equipped with a motion sensor that detected head entries (nosepokes) and was connected to a pellet dispenser located between the two levers. The pellet dispenser dispensed 45 mg reward pellets (Dustless Precision Pellets^®^, Rodent Purified Diet, Bio-Serve, Frenchtown, NJ). A light was mounted above the trough (trough light). A house light was mounted on the opposite wall near the ceiling. The chambers were equipped with shock generators that applied a scrambled current to the grid rod floor. All of the accessories within the chamber were from Med Associates (St. Albans, VT). The chambers were fully enclosed within sound- and light-attenuating boxes (Med Associates, St. Albans, VT), which were equipped with exhaust fans (for air circulation and low background noise) programmed to run during all stages of shaping and testing. The chambers were cleaned before and after each use with a 5% vinegar solution.

Operant chamber programs were written with TRANS IV software (Informatics Inc., Woodland Hills, CA). Programs were run with MED-PC IV software (Med Associates, St. Albans, VT) via lab computers. The software also allowed for recording of events occurring within the chamber (e.g. lever presses, nosepokes, lights turning on/off) and the time at which they occurred.

### Animals and Dosing

**Figure 1:**
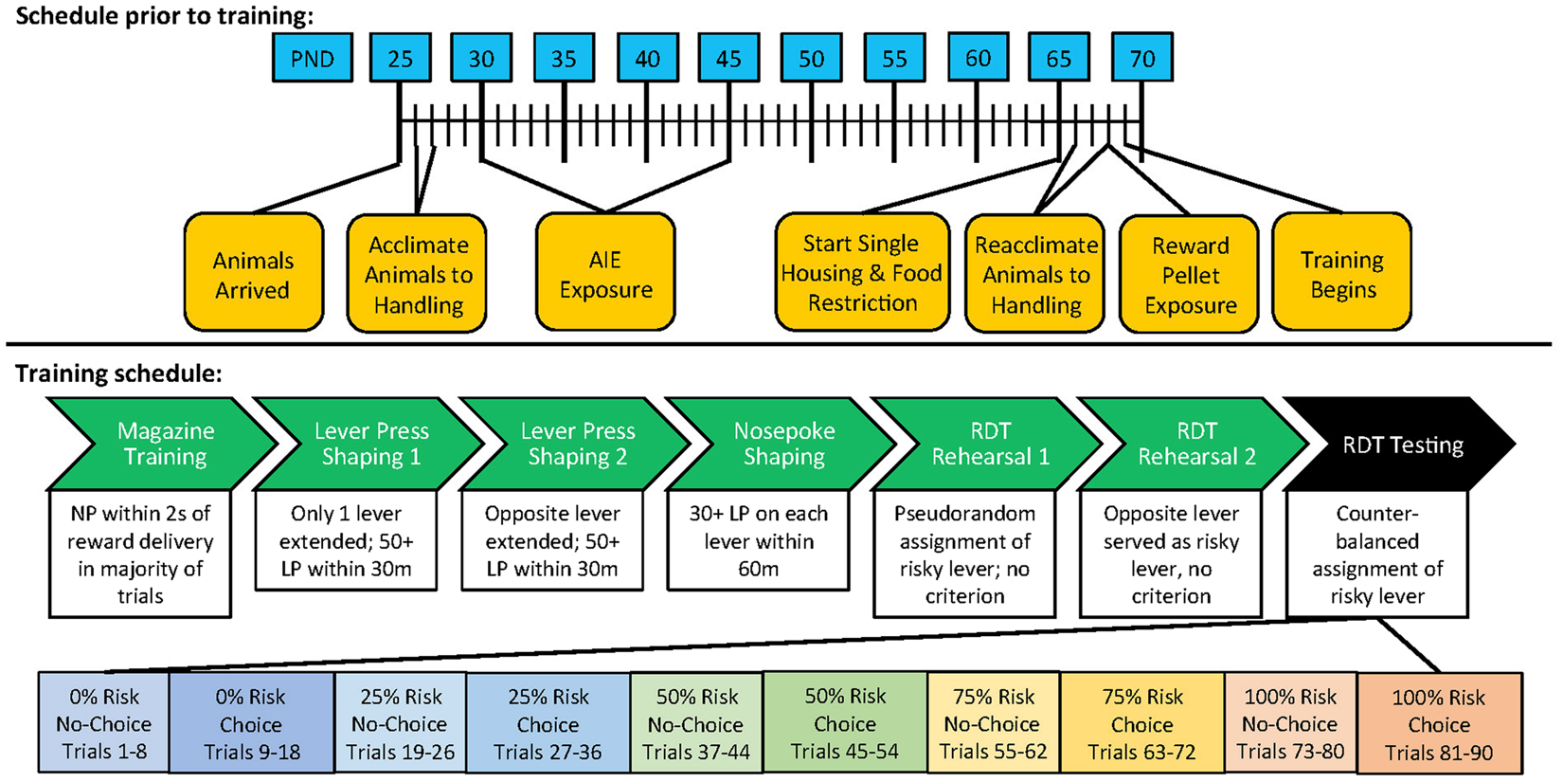
Treatment and training outline.

All procedures were conducted in accordance with protocols approved by the Durham VAMC and Duke University Institutional Animal Care and Use Committees. A total of 16 adolescent male Sprague-Dawley rats (Charles River, Raleigh, NC) were dual-housed on a normal 12:12 hour light:dark cycle (lights on at 6:00 am) with *ad libitum* access to chow and water. Animals were acclimated to handling for 2 days. Beginning on postnatal day (pnd) 30, animals were exposed to either an adolescent intermittent ethanol (AIE) paradigm consisting of 10 doses of 5 g/kg ethanol (35% v/v in saline at 18.12 mL/kg), or isovolumetric saline. Ethanol or saline was administered once/day via intragastric gavage (IG) in a 2-days on, 1-day off, 2-days on, 2-days off sequence such that animals received doses of ethanol or saline on dosing days 1 and 2, 4 and 5, 8 and 9, 11 and 12, 15 and 16 (as described previously^13^). Rats were allowed to reach adulthood before task training took place. See Fig. 1 for a schematic representing this timeline.

### Handling, Food-Restrictive Diet, and Pellet Exposure

On pnd 65 (20 days after the last ethanol/saline dose), rats were moved into individual housing with additional enrichment and were started on a food-restrictive diet, decreasing the animals to 85% of their free-feeing weight over the course of the week. They were maintained at this weight throughout behavioral testing. Water continued to be freely available except during acclimation, training, and testing sessions.

Each rat was habituated to the behavioral researchers via daily five-minute handling sessions from pnd 66–68. During handling sessions, rats were presented with chow so they could associate researchers with food. On pnd 68, rats were introduced to the reward pellets used in the behavioral task during handling in order to reduce neophobia. An additional four pellets were left in each animal’s cage overnight for additional exposure. See Fig. 1 for a schematic depicting this timeline.

On pnd 69, rats began training for the behavioral task. Each day prior to training and testing sessions, animals were weighed and given 30 minutes to acclimate to the testing room. Animals were given their daily chow following training/testing completion.

### RDT Training

Each animal was required to complete multiple stages of testing to criteria before beginning the RDT task. Training included: magazine training, lever press shaping, nosepoke shaping, and RDT rehearsal (modified from Simon’s original task^12, 14^). If an animal “failed” any part of the training, it was repeated until it reached criterion. See Fig. 1 for a schematic representing the training sequence and parameters during training.

#### Magazine Training

Beginning on pnd 69, animals were trained to associate the food trough with reward pellets. Training consisted of 38 trials per session, and there was one session per day. Throughout this shaping, both the house and trough lights were on and the levers were retracted. In each trial, one pellet was dispensed, followed immediately by a randomly generated inter-trial interval (ITI) of duration 100 ± 40 s. A rat met criterion if it performed a nosepoke (resulting from the animal inserting his head into the trough to retrieve the pellet) within two seconds of the reward being dispensed in at least half of the trials.

#### Lever Press Shaping

Animals learned that lever presses resulted in delivery of reward pellets into the trough. Shaping involved at least two days of training: one day for learning each lever. Which levers the animals learned first and second were counterbalanced between treatment groups. Throughout each session, both the house and trough lights were on and only the lever being learned was extended. When an animal pressed the extended lever, one reward pellet was dispensed. Each training session lasted 30 minutes. A rat met criterion if it pressed the extended lever at least 50 times within the time allotted.

#### Nosepoke Shaping

Animals learned that a nosepoke into the trough resulted in lever extension. Lever extensions were generated pseudorandomly, so there were an equal number of extensions of each lever and no lever was extended more than two consecutive times. Each session lasted 60 minutes and there was one session per day. Each session had varying numbers of trials due to the random ITI duration (30–50 s). Each trial started with the house and trough lights on and levers retracted. A nosepoke within the first 10 seconds caused the trough light to turn off and one lever to extend while the house light remained on. The animal then had 10 seconds in which to press the lever. A lever press resulted in the house light turning off, the lever being retracted, and the trough light turning on accompanying the dispensing of one reward pellet. The trough light turned off after the subsequent nosepoke (performed while retrieving pellet) or after another 10 seconds, whichever came first, prompting the initiation of the ITI. At the conclusion of the ITI, the trough and house lights were illuminated and the next trial began.

If the animal failed to lever press in the allotted time or failed to nosepoke to retrieve the pellet, the trial was terminated and the ITI began. A rat met criterion if it completed a minimum of 60 lever presses (at least 30 on each lever) within the 60 min session.

#### RDT Rehearsal

This training functioned as a “run through” for the final RDT (described below). Rats were pseudorandomly (counterbalanced across treatment groups) assigned a large reward lever (e.g. left lever) and a small reward lever (e.g. right lever). A large reward lever press resulted in a large reward (3 reward pellets) and a small reward lever press resulted in a small reward (1 reward pellet) being deposited into the trough. The rehearsal consisted of 18 trials: 8 “no-choice” trials followed by 10 “choice” trials. “No-choice” trials involved the presentation of only one of the two levers and taught the rats that pressing different levers resulted in differently sized rewards. “Choice” trials involved the extension of both levers simultaneously and allowed the animal to choose which lever to press (if any).

As in the nosepoke shaping, these trials started with both the house and trough lights on and the levers retracted. Rats were given 10 seconds to respond with a nosepoke. Nosepoking extinguished the trough light and ensured that rats were centered between the levers to prevent positional bias. In “no-choice” trials, a nosepoke resulted in one lever being extended. In “choice” trials, a nosepoke resulted in both levers being extended. Following a lever press, the trough light was re-illuminated to signal that reward pellets were delivered to the trough (1 pellet for the safe lever and 3 pellets for the risky lever), the house light was extinguished and the levers were retracted. The trough light remained on for 10 seconds or until the animal completed a nosepoke (by retrieving the reward), whichever came first. Once the trough light was extinguished, the ITI began (20 s).

Failure to complete the trial’s initial nosepoke or a lever press in the times allotted resulted in both house and trough lights being extinguished, levers retracted (if out), and the trial being terminated, triggering the start of the ITI. Trials without completed initial nosepokes were counted as “omissions” and trials without completed lever presses were counted as “partial omissions”.

Animals were tested on the RDT Rehearsal for 2 days, one session per day. The large reward and small reward levers were reversed for the second session to help prevent development of bias towards one lever prior to testing.

### Risky Decision-Making Task (RDT)

Rats were randomly (in a counterbalanced manner across treatment groups) assigned a risky, large reward lever (e.g. the right lever) and a safe, small reward lever (e.g. the left lever), and that lever choice was kept constant throughout testing. A risky lever press resulted in a large reward (3 reward pellets) and could also result in a 0.35 mA footshock (current determined through pilot testing, data not shown) of varying duration (1.0, 1.2, 1.3, or 1.5 s). The duration of the shock was varied to prevent shock tolerance. A safe lever press resulted in a small reward (1 reward pellet) and no risk of footshock.

Each RDT testing session lasted approximately 60 minutes and consisted of five blocks of 18 trials. Each block presented subjects with a different level of risk of footshock: 0%, 25%, 50%, 75%, or 100% risk. Risk levels were always presented in ascending order to eliminate the influence of “re-learning” of the task. Of the 18 trials, the first 8 were “no-choice” trials and the next 10 were “choice” trials.

“No-choice” trials involved the presentation of only one lever and served to model the risk level of the block to the rat (i.e., the probability of getting a shock when choosing the risky lever). Levers were presented in a pseudorandom manner in these trials (no more than two extensions of the same lever in a row) and risk was associated with risky lever presses in set ratios. For example, in the 25% risk block, exactly one in every four risky lever presses would be followed by a shock.

“Choice” trials involved the extension of both levers simultaneously and allowed the animal to demonstrate preference for risk. Risk of shock following a risky lever press was determined by a random number generator selecting a number that was either associated with shock or no shock. For example, in the 25% risk block, there was a 1 in 4 chance (25% risk) of being shocked after a risky lever press.

As in the RDT Rehearsal, these trials started with both the house and trough lights on and the levers retracted. Rats were given 10 seconds to respond with a nosepoke. Nosepoking extinguished the trough light and ensured that rats were centered between the levers to prevent positional bias. In “no-choice” trials, a nosepoke resulted in one lever being extended. In “choice” trials, a nosepoke resulted in both levers being extended, and the animal was allowed to choose which lever to press (if any). Following a lever press: the trough light was re-illuminated to signal that reward pellets were delivered to the trough (1 pellet for the safe lever and 3 pellets for the risky lever); the footshock risk was calculated (if any) and administered as needed; the house light was extinguished; and the levers were retracted. The trough light remained on for 10 seconds or until the animal completed a nosepoke (by retrieving the reward), whichever came first. The trough light remained on for 10 seconds or until the animal completed a nosepoke (by retrieving the reward), whichever came first. Once the trough light was extinguished, the ITI began (20 s).

**Figure 2:**
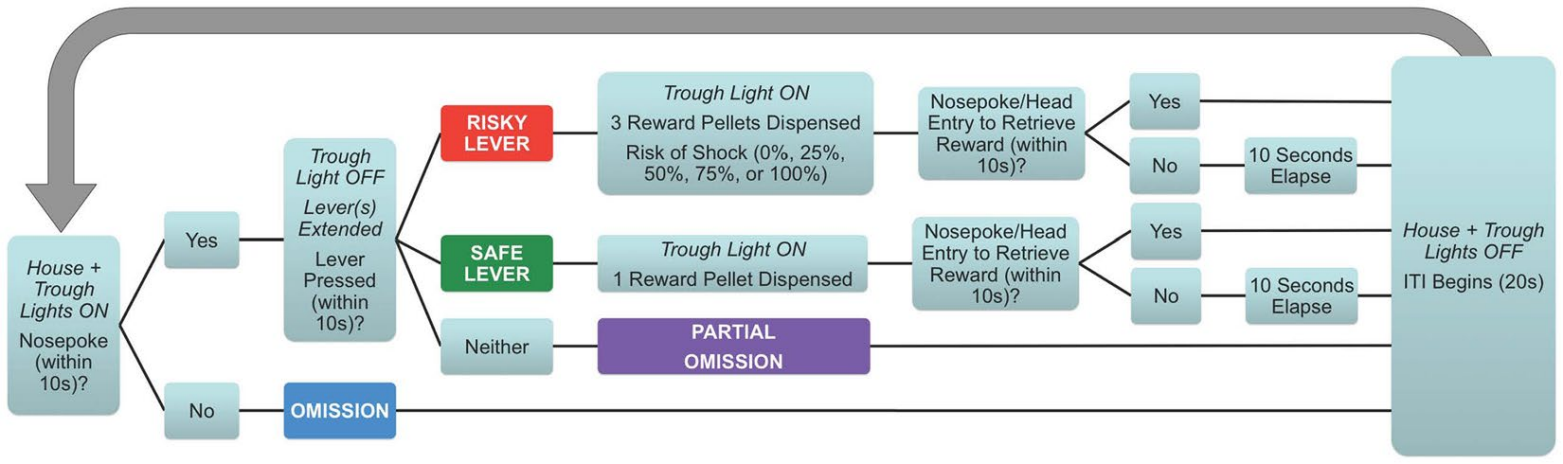
Risky decision-making task (RDT) outline.

Failure to complete the trial’s initial nosepoke or a lever press in the times allotted resulted in both house and trough lights being extinguished, levers retracted (if out), and the trial being terminated, triggering the start of the ITI. Trials without completed initial nosepokes were counted as “omissions” and trials without completed lever presses were counted as “partial omissions.” See Fig. 2 for an outline of each trial’s sequence. Animals were tested on the RDT for four weeks (20 days of testing).

The following parameters were measured and analyzed for each risk level (0%, 25%, 50%, 75%, 100%):

1. Latency to Initial Nosepoke (in no-choice, choice trials) = food motivation/gross motor movement = time elapsed between onset of trough light stimulus and initial nosepoke (if initial nosepoke made).
2. Latency to Lever Press Overall (in no-choice, choice trials) = processing speed = time elapsed between extension of levers and lever press (if lever press made, and regardless of lever press choice).
3. Latency to Risky Lever Press (in no-choice, choice trials) = processing speed = time elapsed between extension of lever(s) and lever press (if risky lever press made).
4. Latency to Safe Lever Press (in no-choice, choice trials) = processing speed = time elapsed between extension of lever(s) and lever press (if safe lever press made).
5. Risky Lever Presses (in no-choice, choice trials) = number of risky lever presses (max 4 per risk level per day in no-choice trials; max 10 per risk level per day in choice trials).
6. Safe Lever Presses (in no-choice, choice trials) = number of safe lever presses (max 4 per risk level per day in no-choice trials; max 10 per risk level per day in choice trials).
7. Omissions (in no-choice, choice trials) = number of trials in which subject does not perform the initial nosepoke within the appropriate window, causing trial termination.
8. Partial Omissions (in no-choice trials, in no-choice trials in which risky lever extended, in choice trials) = number of trials in which the subject does not press an extended lever within the appropriate window, causing trial termination.

### Hot Plate Test

Upon completion of the four weeks of RDT, animals were tested for differences in foot sensitivity using a hot plate test. This test was conducted to determine if AIE animals differed from controls in pain sensitivity, because a difference could confound the interpretation of the results in our RDT task that is motivated, in part, by foot-shock avoidance. An analgesia hot plate (Columbus Instruments, Columbus, OH) was set up in the testing room. Animals were habituated to the testing room for 30 minutes. The hot plate was heated to 55 **°**C and animals were placed individually on the plate. A researcher timed how long the animal stood on the hot plate before showing any signs of discomfort (e.g. licking a paw, flicking its tail). The animal was then removed from the hot plate and placed back in his home cage. After 20 minutes, the hot plate test was repeated. The hot plate was washed with a 5% vinegar solution between usages.

### Data Analysis

Statistical analyses were executed using SPSS v23 and v24 (IBM, Armonk, NY). The following behavioral parameters were analyzed in both choice and no-choice trials.

#### Risky lever presses

The mean number of risky lever presses during choice trials was the primary dependent measure. These data were analyzed using a RM-ANOVA with risk level as the repeated measure and treatment group as the independent variable. Degrees of freedom in the RM-ANOVA were adjusted using the Greenhouse-Geisser procedure when the sphericity assumption was not met. We looked at this measure across a 5-day window following animal reaching stability. An animal was determined to have reached stability if its daily risky lever pressing behavior at the 50% risk level varied by no more than 1 lever press across 5 consecutive days. RM-ANOVA was used in an effort to demonstrate task validity. Consistent with previously published work by other laboratories, we anticipated a trend toward fewer risky lever presses as the risk level increased.

We also analyzed the mean number of risky lever presses during no-choice trials across 5 days post-stability via RM-ANOVA.

#### Number of safe lever presses

The mean number of safe lever presses was another way to look at the animals’ risky decision-making. Mean numbers of safe lever presses in choice and no-choice trials were analyzed using RM-ANOVA. These data were taken from the 5 days post-stability (as determined by risky lever pressing behavior, above).

#### Number of omissions

Omissions data were analyzed to observe subject’s participation. The mean numbers of omissions in both choice and no-choice trials were analyzed using RM-ANOVA. These data were taken from the 5 days post-stability (as determined by risky lever pressing behavior, above).

#### Number of partial omissions

Partial omissions data were analyzed to observe subject’s participation. The mean number of partial omissions in choice trials, no-choice trials, and no-choice trials in which the risky lever was the only lever extended were analyzed using RM-ANOVA. These data were taken from the 5 days post-stability (as determined by risky lever pressing behavior, above).

#### Latency to initial nosepoke, lever press

Latency to nosepoke and latency to lever press (latency to overall lever press, risky lever press, and safe lever press) for both choice and no-choice trials were analyzed using MANOVA with measures at each risk level being separate dependent variables and treatment group as the independent variable. These data were taken from all 20 days of RDT performance. Both latency to lever press (overall, risky, and safe) and latency to nosepoke are used as measures of general behavioral performance. As such, we were interested in these variables across all days. This is in contrast to some of the other measures analyzed (e.g., the number of risky lever presses, which is used as a task specific measure of behavior). Moreover, RM-ANOVA was not used because we did not anticipate changes in these global behavioral characteristics as a function of risk level. MANOVA was used to control for multiple comparisons by evaluating the group effect on latency (to lever press or nosepoke) at each of the risk levels simultaneously. Significant multivariate outcomes were followed with univariate analyses. However, this approach was not possible for analyses of the latency to risky lever press in choice trials. Not all animals responded with at least one risky lever press at each risk level block, especially at the higher risk levels. Use of MANOVA would have required deletion of any animal for which there was no data at any risk level. This would have resulted in the loss of three saline pre-treated animals and four ethanol pre-treated animals. To better preserve our sample size we used independent sample t-tests on those data and applied a Bonferroni correction to our alpha to adjust for multiple comparisons (alpha = 0.01).

#### Hot plate response time

Response time on the hot plate test was assessed using a Student’s independent sample t-test.

#### Statistics

Due to the directional nature of our hypotheses, F statistic p-values corresponding to the group effect (AIE vs. control) were adjusted by a factor of 0.5. We sought to test directional hypotheses concerning the effect of our independent variable (AIE vs. control) on 5 separate dependent variables (e.g., latency to lever press at 0%, 25%, 50%, 75% and 100% risk). That is, we made one-tailed hypotheses regarding the relative performance of the AIE animals compared to control animals based on the specific nature of the dependent variable in question. Although we have only two levels of our IV (AIE vs. control), we also had 5 dependent variables – performance at each level of risk. To control for experiment-wise error, we employed a multivariate ANOVA (MANOVA) where the IV was group (AIE vs. control) and the DVs were performance at each level of risk. While use of MANOVA reduces the experiment-wise error associated with multiple DVs, F tests such as those used in MANOVA are inherently non-directional.

While it is generally understood that F distributions are one tailed, it is often overlooked that they are also non-directional. F-tests such as those used in MANOVA are usually reserved for situations in which the independent variable (IV) consists of three or more levels. As such, it is rare for investigators to make specific hypotheses about the particular directions of the observed outcomes. When such predictions are made, planned comparisons are typically used following a significant F value to test specific hypotheses about differences between levels of the IV. With only two levels of the IV in our study (AIE vs. control), the observed probability corresponding to the F value covers all outcomes: AIE >control and control >AIE. In fact, when only two levels are present within an IV, the corresponding F value will be equivalent to the square of the Student’s T value collected on the same data. Moreover, the p value associated with the F will be equal to the two-tailed probability associated with the Student’s T value. To make the probability directional (i.e., one-tailed), we simply divided the p-value by 2.

## Results

### Risky Lever Presses

**Figure 3:**
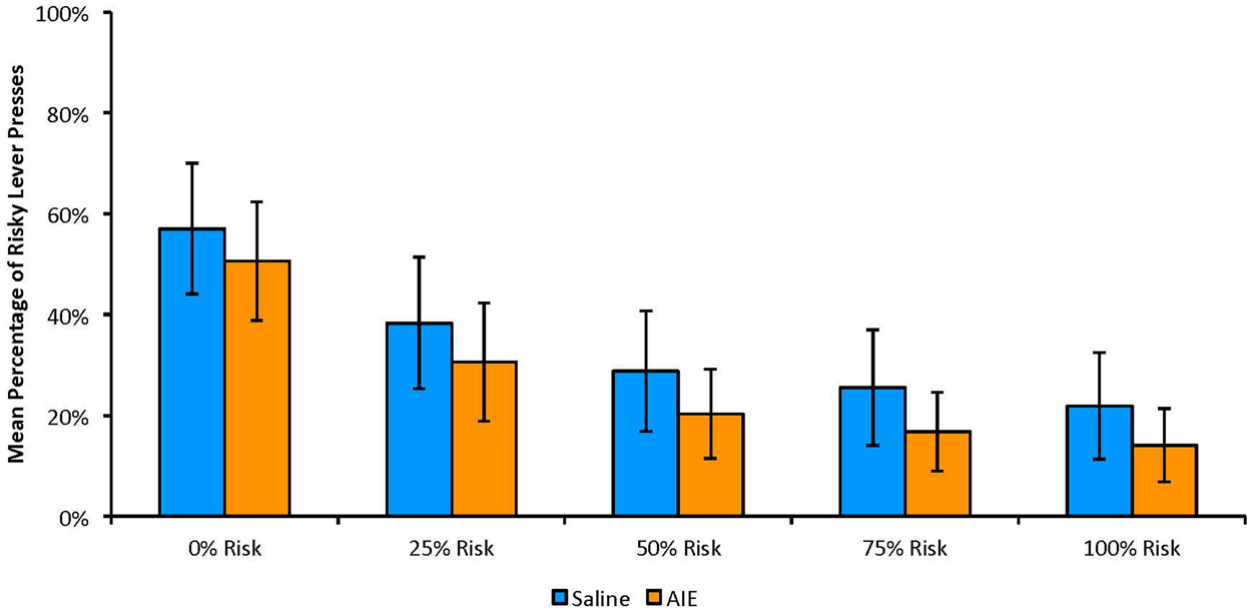
Mean (±SEM) percentage of risky lever presses in choice trials at each risk level, averaged across the 5 days post-stability for each animal. No significant treatment effect found (p’s > 0.01).

As expected, the number of risky lever presses decreased as the probability of footshock increased (choice trials: F(4,56) = 10.15, p ≤ 0.001; no-choice trials: F(4,56) = 38.83, p ≤ 0.001), demonstrating validity of the overall behavioral model (Fig. 3). However, there was no effect of AIE on the number of risky lever presses (choice trials: F(1,14) = 0.482, p = 0.50; no-choice trials: F(1,14) = 0.027, p = 0.87) and there was no interaction of group and risk level (choice trials: F(4,56) = 0.52, p = 0.72; no-choice trials: F(4,56) = 2.02, p = 0.6).

### Safe Lever Presses

The number of safe lever presses increased as the probability of footshock increased in choice trials (F(4,56) = 10.34, p = 0.003), but not in no-choice trials (F(4,56) = 1.03, p = 0.33). There was no effect of AIE on the number of safe lever presses (choice trials: F(1,14) = 0.45, p = 0.51; no-choice trials: F(1,14) = 0.92, p = 0.35), and there was no interaction of group and risk level (choice trials: F(4,56) = 0.45, p = 0.55; no-choice trials: F(4,56) = 0.85, p = 0.38).

### Omissions

Few omissions occurred in either choice or no-choice trials, and as a result, we had insufficient data to analyze. In choice trials, one saline- and one AIE-treated animal omitted trials at the 0% and 100% risk levels, and two other AIE animals omitted choice trials at the 75% risk level. In no-choice trials, two AIE animals at the 50% risk level and one AIE animal at the 100% risk level were observed to omit trials. No saline-treated animals omitted any no-choice trials at any risk level.

### Partial Omissions

Few partial omissions occurred in choice trials and thus we had insufficient data to analyze. One saline-treated animal partially omitted choice trials in the 0% risk level, and another partially omitted choice trials in the 75% risk level. Two AIE animals partially omitted choice trials in the 0% risk level, one partially omitted choice trials at the 50% risk level, and a fourth AIE animal partially omitted choice trials in both the 75% and 100% risk levels. In no-choice trials, the number of partial omissions increased as risk of footshock increased (no-choice trials: F(4,56) = 11.92, p ≤ 0.001; no-choice trials with risky lever extended: F(4,56) = 12.07, p ≤ 0.001). There was no effect of AIE on the number of partial omissions (no-choice trials: F(1,14) = 0.015, p = 0.9; no-choice trials with risky lever extended: F(1,14) = 0.038, p = 0.85), and there was no interaction of group and risk level (no-choice trials: F(4,56) = 0.54, p = 0.57; no-choice trials with risky lever extended: F(4,56) = 0.48, p = 0.6).

### Latency to Lever Press

**Figure 4:**
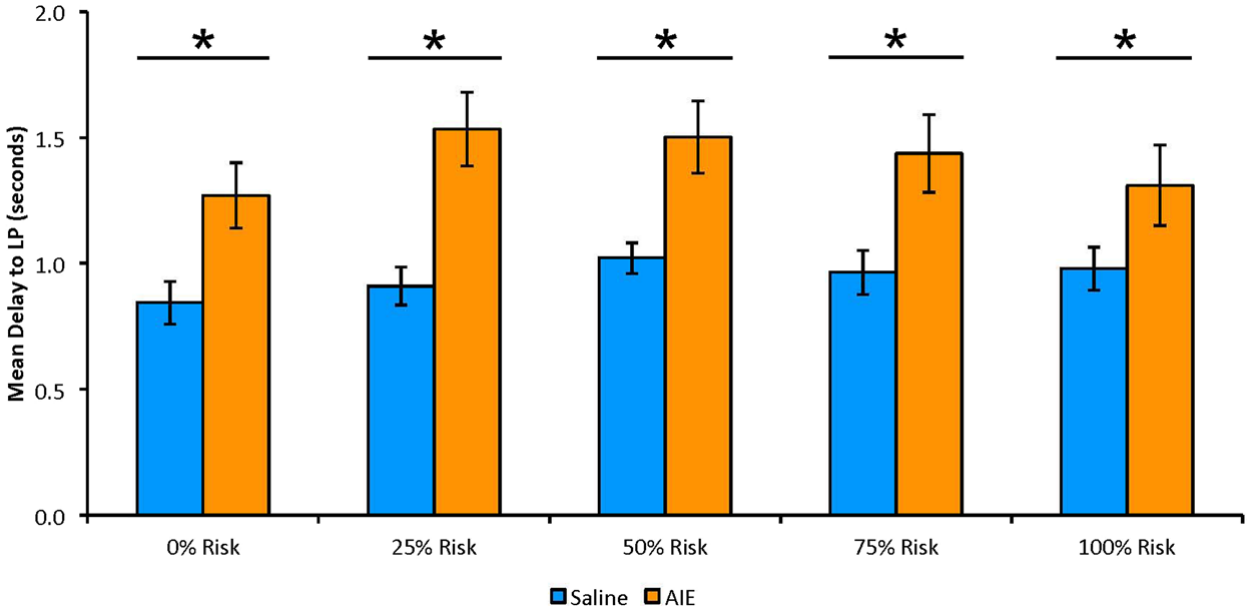
Mean (±SEM) delay to lever press (LP), regardless of lever choice, in choice trials at each risk level, averaged across all days of testing (days 1–20). Maximum potential delay to lever press is 10 s. AIE results in increased delay time at each of the 5 risk levels. * = p ≤ 0.01. Statistics: Multiple t-tests, Bonferonni correction −0% Risk: F(1,14) = 7.52, p = 0.008; 25% Risk: F(1,14) = 14.34, p = 0.001; 50% Risk: F(1,14) = 9.48, p = 0.004; 75% Risk: F(1,14) = 7.13, p = 0.009; 100% Risk: F(1,14) = 3.31, p = 0.045.

AIE markedly increased the latency to lever press across choice trials, regardless of the choice made, F(5,10) = 2.79, p = 0.04 (Fig. 4). Follow-up univariate tests reveal significant AIE-induced increases in response time at each risk level [0%: F(1,14) = 7.52, p = 0.008; 25%: F(1,14) = 14.34, p = 0.001; 50%: F(1,14) = 9.48, p = 0.004; 75%: F(1,14) = 7.13, p = 0.009; 100%: F(1,14) = 3.31, p = 0.045]. However, these results were not found in no-choice trials, F(5,10) = 1.74, p = 0.21. There was no difference between treatment groups in the latency to risky lever press (choice trials: [0%: t(14) = −1.77, p = 0.05; 25%: t(14) = −0.36, p = 0.37; 50%: t(12) = −1.02, p = 0.16; 75%: t(10) = −1.67, p = 0.07; 100%: t(7) = −0.42, p = 0.35]; no-choice trials: F(5,10) = 0.68, p = 0.65). There was also no difference between treatment groups found in the latency to safe lever press (choice: F(5,10) = 2.50, p = 0.102; no-choice: F(5,10) = 1.22, p = 0.37).

### Latency to Initial Nosepoke

**Figure 5:**
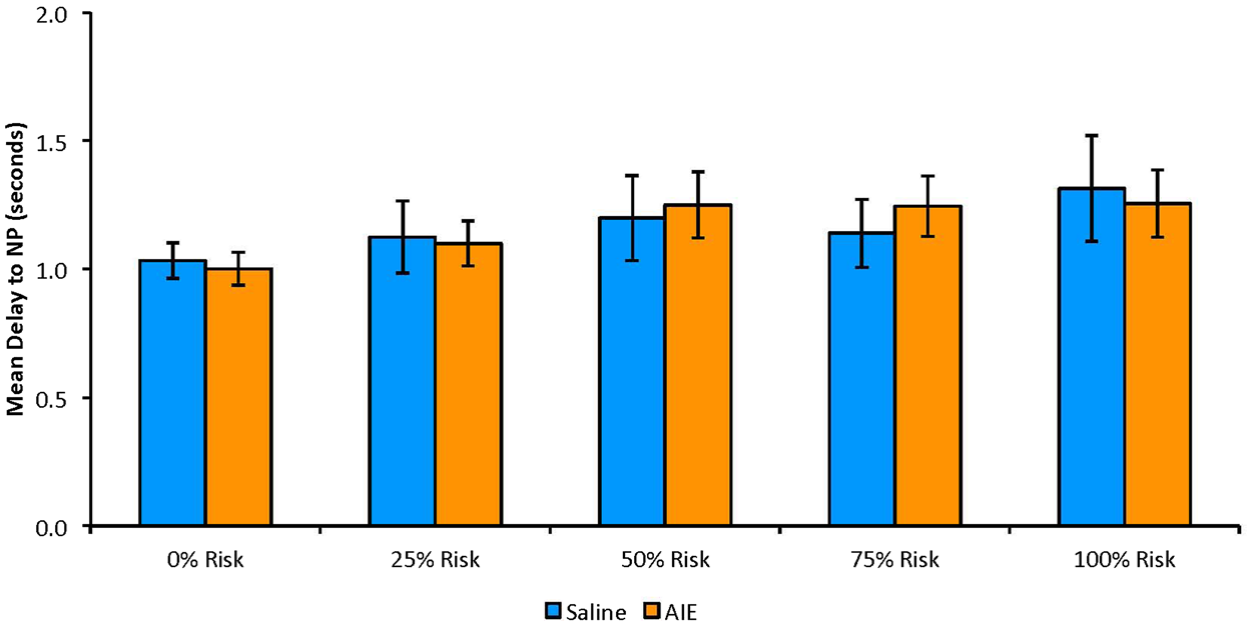
Mean (±SEM) delay to initial nosepoke (NP) in choice trials at each risk level, averaged across all days of testing (days 1–20). Maximum potential delay to nosepoke is 10 s. No difference found between treatment groups.

Although AIE increased the latency to lever press, it did not alter the latency to initial nosepoke, (choice trials: F(5,10) = 1.29, p = 0.17, Fig. 5; no-choice trials: F(5,10) = 0.18, p = 0.97), suggesting that there was no difference in general behavioral response time between AIE and control animals.

### Hot Plate Test

**Figure 6:**
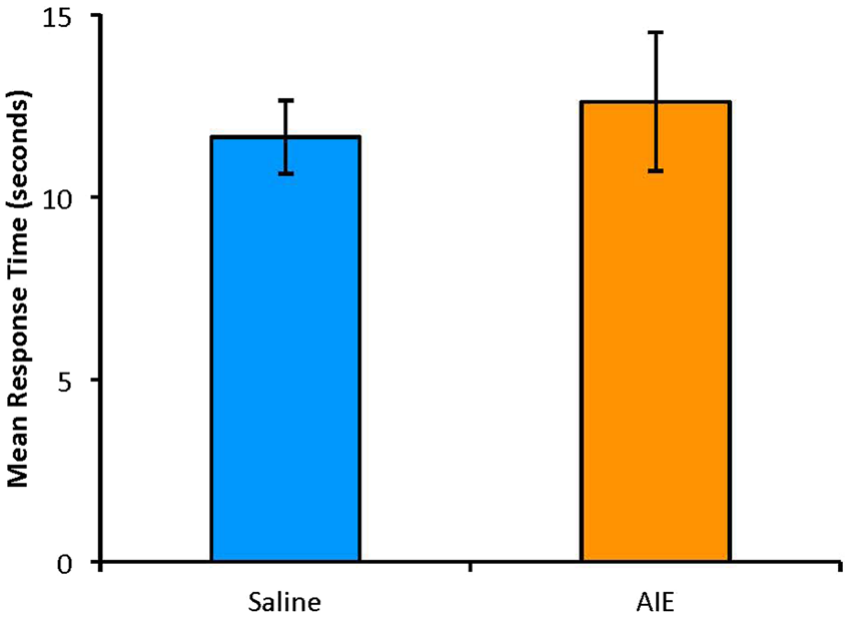
Mean (±SEM) response times on hot plate test (55 **°**C), ±SEM. No significant difference between treatment groups.

There was no difference in nociceptive sensitivity between control rats and those previously exposed to AIE, as assessed using the hot plate test, t(14) = −0.45, p = 0.66 (Fig. 6).

## Discussion

The principle findings in this study are that the latency to lever pressing was significantly increased among AIE animals compared to controls, and that AIE did not produce a long term change in punishment-mediated risky decision making. The AIE effect on latency to lever pressing does not appear to be the result of gross motor impairment, as there was no difference in latency to initial nosepoke between AIE animals and controls. Previous studies from our laboratory are consistent with this, as we have not observed AIE-induced changes in motor performance speed in either radial arm^15^ or water maze^13^ tasks or on operant performance^15^. The delay to lever press is similarly unlikely to be due to differences in food motivation, as we have found no differences in food motivation between AIE exposed animals and controls^15^.

Although we had not anticipated the AIE-induced delay in lever pressing, it is consistent with previous studies in which the behavioral efficiency of animals exposed to AIE has been compromised. For example, we have shown that the time to execute a choice in the Morris water maze task in adulthood was compromised by AIE, although AIE did not affect spatial or non-spatial learning directly in that task^13^. Because both the RDT and the water maze task are negatively-motivated (punishment and negative reinforcement, respectively), it is possible that AIE is more likely to compromise behavioral efficiency in negatively-motivated, rather than appetitively-motivated tasks. Consistent with this interpretation, we have not observed behavioral inefficiency in AIE-exposed animals on food-motivated maze or operant tasks^15^. Therefore, it is likely that the deficits we observed in the latency to lever press were driven by AIE-induced compromise of behavioral efficiency related to processing speed under the stress of an aversively motivated task. At present it is not clear if AIE alters subsequent stress responsiveness, but it is a plausible hypothesis and represents an intriguing possible mechanism underlying the present findings on response latency. Further, it is noteworthy that one of the principal compromises of cognitive function in human adolescents after a history of repeated, heavy alcohol exposure is decreased psychomotor speed^16^, which reflects a compromise of behavioral efficiency on neuropsychological assessments. In general, behavioral efficiency refers to the relationship between accuracy of responding and the time required to form the response. Humans and other animals behave most efficiently when they are working as quickly as they can without making mistakes. Cognitive or behavioral efficiency becomes biased when one of two outcomes occur: 1) work occurs quickly at the expense of accuracy, or 2) work occurs accurately at the expense of speed. Moreover, cognitive or behavioral efficiency generally refers to situations in which a decision must be made between competing stimuli. For example, choosing the risky lever or the safe lever in the choice trials. This is often contrasted with simple reaction time, which typically reflects impulsivity in the absence of competing stimuli.

It was somewhat surprising that we found no significant effect of AIE on risk taking in the present task (although the level of risk clearly influenced lever-pressing choices in both AIE-exposed animals and controls). Our finding appears inconsistent with those from other groups that have used probability-discounting tasks to measure risky decision-making in rodents^6–9^. However, the risk in those studies was of a decreased probability of reward on a given trial, not on an increased probability of punishment, as in the present study. The presence of differing mechanisms underlying probabilistic vs. punishment-based decision-making is well documented. As reviewed by Orsini *et al*., the two types of decision making involve D2-like dopamine signaling, the basolateral amygdala (BLA) and the orbitofrontal cortex (OFC), yet with opposite effects^17^. For example, stimulation of D2-like receptors increases the choice of the risky reward in probability-based decision-making, but decreases choice of risky reward in punishment-based decision-making. Similarly, lesioning of the BLA decreases choice of the risky reward in probability-based decision-making, but increases risky choice in punishment-based decision-making. Finally, lesioning of the OFC increases choice of the risky reward in probability-based decision-making, but decreases risky choice in punishment-based decision-making. These differences seem to be attributable to whether the neurons involved encode salience or action, and work is ongoing in this area. For our purposes, therefore, given the distinct dissociation between neural representations of appetitive and aversive stimulus values^18^, it is not surprising that our results in a punishment-based task would differ from prior results in probability-based tasks, differently motivated tasks would result in distinctly different outcomes, particularly when measuring the long-term effects of a treatment applied during adolescence.

Clearly, risk parameters are an important determinant of risk taking. The present findings underscore the need for precisely defined and understood parameters in future studies, particularly when those studies endeavor to use models of risky decision-making to assess the effects of drug or toxicant exposures. Beyond those parametric considerations, however, the present study adds to a growing literature, in both animal models and humans, which indicates an AIE-induced reduction in behavioral efficiency that persists into adulthood. Future studies will be designed to assess the persistence of this AIE-induced behavioral deficit across adulthood, or perhaps hasten the onset of age-related behavioral declines. It will also be valuable to determine whether risky decision-making among adolescents is driven differently by risk mediated by the likelihood of appetitive vs. aversive stimuli.
